# In This Issue

**Published:** 2002

## GENETIC STRATEGIES TO DETECT GENES INVOLVED IN ALCOHOLISM AND ALCOHOL-RELATED TRAITS

Developments in genetic technologies are allowing scientists to better study complex disorders such as alcoholism. As described by Drs. Danielle M. Dick and Tatiana Foroud, these strategies include using linkage approaches to identify genes or gene variants that occur more commonly in people with alcoholism than in people without the disorder. Alternatively, researchers can conduct candidate gene analyses that explore the association between a particular candidate gene (which has been identified based on other studies) and the disorder. Genetic studies using appropriately bred strains of laboratory animals also can help identify human genes involved in alcoholism. The authors discuss the clinical implications of a variety of technologies, including genetic counseling, gene therapy, and the development of new medications based on genetic discoveries. (pp. 172–180)

## GENOMIC APPROACHES TO THE GENETICS OF ALCOHOLISM

Although researchers have deciphered the entire human genetic material (i.e., the genome), they still must determine which of those sequences actually represent genes, which have regulatory functions, and which cannot yet be assigned a specific function. As Drs. Marissa A. Ehringer and James M. Sikela report, numerous computer-based analytical methods are now available to assist investigators in determining the functions of genetic sequences, which is also called annotation. For example, bioinformatics and annotation tools can help researchers identify sequences that are similar to known genes, sequence motifs that signal the start or end of a gene, or motifs with known regulatory functions. Numerous public and private resources are available to provide such tools. Other tools, such as gene chips, allow investigators to identify genes that are active in a given cell at a given time. The potential applications of these genomic approaches to alcohol research only now are beginning to emerge. (pp. 181–192)

## EFFECTS OF THE INTERACTION BETWEEN GENOTYPE AND ENVIRONMENT: RESEARCH INTO THE GENETIC EPIDEMIOLOGY OF ALCOHOL DEPENDENCE

Each person’s risk of alcohol dependence is determined by the interplay between genetic and environmental risk factors. The interactions between a person’s genetic makeup and his or her environment are called “genotype × environment” (GxE) interactions. Drs. Andrew C. Heath and Elliot C. Nelson explore the relationships between GxE interactions, alcohol dependence, and co-occurring disorders such as depression. Specific family study approaches, such as research using children of twins, may help clarify these relationships and the extent of GxE interaction effects. Molecular epidemiologic studies using case control and prospective cohort approaches must consider the potential role of GxE interaction effects in their designs to obtain meaningful results. (pp. 193–201)

## ANIMAL MODELS FOR THE GENETIC STUDY OF HUMAN ALCOHOL PHENOTYPES

Animal models can be used to identify novel genes that may contribute to the development of alcoholism in humans and to further extend genetic discoveries made in humans, particularly because researchers can tightly control the genetic and environmental characteristics of the animals being studied. Dr. Tamara Phillips reviews some of the techniques used in these studies, including the use of knockout mice, in which the function of a specific gene has been disrupted, and transgenic mice, in which a foreign gene has been introduced. A technique called QTL mapping, combined with well-controlled breeding schemes, allows researchers to identify the regions of specific chromosomes that harbor genes influencing certain traits. Other techniques, such as random mutagenesis or virus-mediated gene transfer, also are becoming more important for studies in rodents as well as in other species (e.g., fruit flies, zebrafish, and nonhuman primates). (pp. 202–207)

## DEFINING ALCOHOL-RELATED PHENOTYPES IN HUMANS: THE COLLABORATIVE STUDY ON THE GENETICS OF ALCOHOLISM

The Collaborative Study on the Genetics of Alcoholism (COGA) is an innovative, large-scale, multidisciplinary research program launched by the National Institute on Alcohol Abuse and Alcoholism to investigate the genetic components that contribute to the development of alcohol abuse and dependence. The study—which involves 9 research centers located across the United States—relies primarily on DNA samples, questionnaires, electrophysiological measurements, and other data obtained from nearly 3,000 people from families with at least 3 alcoholic members. Dr. Laura Jean Bierut and her colleagues describe the genetic analyses conducted on these DNA samples in an effort to identify DNA regions (i.e., loci) that carry genes influencing the risk of alcoholism and other alcohol-related traits. Although focusing on the methodology of the COGA study, the authors also summarize some key findings regarding loci on chromosomes 1 and 4 that influence such traits as alcohol dependence, level of response to alcohol, presence of alcoholism or depression, or maximum number of drinks a person may consume in one sitting. (pp. 208–213)

## THE COLLABORATIVE STUDY ON THE GENETICS OF ALCOHOLISM: AN UPDATE

Researchers participating in the Collaborative Study on the Genetics of Alcoholism (COGA) are systematically screening all human chromosomes for evidence of DNA regions carrying genes that influence the risk of alcoholism and other related traits. This article by Dr. Howard J. Edenberg provides an update on COGA’s findings, including the fact that certain regions on chromosomes 1, 2, 3, and 7 have been identified which may increase a person’s risk of alcoholism. Conversely, genes located on chromosome 4 may have a protective effect. COGA researchers also have identified DNA regions that influence symptoms related to alcoholism, that are associated with disorders commonly co-occurring with alcoholism, or which are linked with certain electrophysiological measures commonly detected in alcoholics. (pp. 214–218)

## PROTEOMICS IN ALCOHOL RESEARCH

Researchers are interested not only in the genes that may contribute to the development of alcoholism but also in the functions and activities of the proteins encoded by those genes. The relatively new field of proteomics—the large-scale analysis of protein structure and function as well as of protein–protein interactions—may allow substantial progress in this endeavor. Drs. Helen Anni and Yedy Israel present some of the techniques used in proteomics analyses, such as two-dimensional gel electrophoresis, high-performance liquid chromatography, and their modifications, as well as potential applications of these technologies in the alcohol field. Together with other sophisticated techniques for studying protein–protein interactions, these approaches eventually will provide valuable information on the mechanisms underlying the development of alcoholism and alcohol-induced organ damage. (pp. 219–232)

## IS THERE A GENETIC RELATIONSHIP BETWEEN ALCOHOLISM AND DEPRESSION?

It has long been known that alcoholism and depression tend to occur together and that both disorders may run in families. Researchers participating in the Collaborative Study on the Genetics of Alcoholism (COGA) have investigated the prevalence of alcoholism and depression in alcoholic participants and their family members. According to Dr. John I. Nurnberger, Jr., and his colleagues, these analyses found that the prevalence of depressive syndrome (i.e., depression that may or may not occur in conjunction with increased drinking) was higher among alcoholics than among nonalcoholics. Moreover, both disorders co-occurred more commonly among family members of people with both disorders than among family members of people with alcoholism alone. (pp. 233–240)

